# Insertion of (Bioactive) Equatorial Ligands into Platinum(IV) Complexes

**DOI:** 10.1002/anie.202311468

**Published:** 2023-10-13

**Authors:** Alexander Kastner, Hemma Schueffl, Patrick A. Yassemipour, Bernhard K. Keppler, Petra Heffeter, Christian R. Kowol

**Affiliations:** ^1^ University of Vienna Faculty of Chemistry Institute of Inorganic Chemistry Waehringer Str. 42 1090 Vienna Austria; ^2^ Center for Cancer Research and Comprehensive Cancer Center Medical University of Vienna Borschkegasse 8a 1090 Vienna Austria; ^3^ Research Cluster “Translational Cancer Therapy Research” 1090 Vienna Austria; ^4^ University of Vienna Vienna Doctoral School in Chemistry (DoSChem) Waehringer Str. 42 1090 Vienna Austria

**Keywords:** Antitumor Agents, Bioinorganic Chemistry, Platinum, Synthesis Design

## Abstract

Platinum(IV) prodrugs are highly interesting alternatives to platinum(II) anticancer therapeutics due to their increased tumor selectivity and reduced side effects. In contrast to the established theory, we recently observed that the equatorial ligand(s) of e.g. oxaliplatin(IV) complexes can be hydrolyzed with formation of [(DACH)Pt(OH_eq_)_2_(OAc_ax_)_2_]. In the work presented here, we investigated the reactivity and synthetic usability of this complex to be exploited as a precursor for the development of novel platinum(IV) complexes, not able to be synthesized by conventional protocols. Indeed, we could substitute the equatorial hydroxido ligand(s) e.g. by one or two monodentate biotin ligands (which would be oxidized under standard methods). The formed complexes turned out to be very stable with slow ligand release after reduction, ideal for long‐circulating tumor‐targeting strategies. Therefore, two platinum(IV) complexes with equatorial maleimides, capable of exploiting serum albumin as a natural nanocarrier, were synthesized as well. The complexes showed massively prolonged plasma half‐life and distinctly improved anticancer activity in vivo compared to oxaliplatin. Taken together, the newly developed synthetic platform allows the simple and specific insertion of equatorial ligands into platinum(IV) complexes. This will enable the attachment of three different (bioactive) moieties generating targeted triple‐action platinum(IV) prodrugs within one single platinum complex.

## Graphical abstract

We investigated the incorporation of equatorial ligands into platinum(IV) complexes, enabling the synthesis of otherwise unavailable complexes. Attachment of albumin‐targeting maleimide moieties led to increased tumor accumulation and anticancer activity compared to oxaliplatin. The presented strategy will allow the development of new types of targeted multi‐action platinum(IV) complexes.

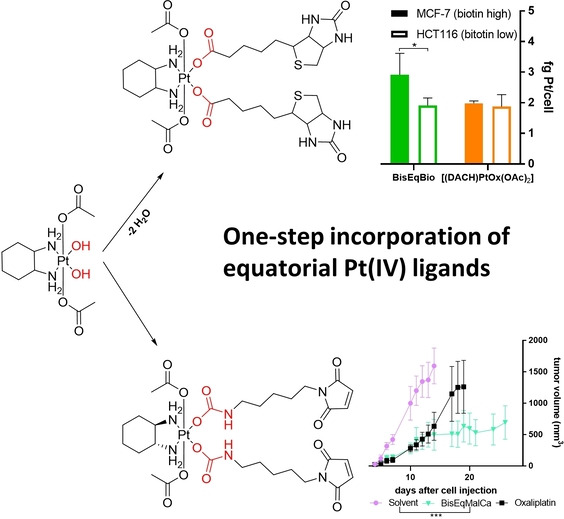

## Introduction

Chemotherapy is still a very important part of cancer treatment. Especially platinum complexes are frequently used, also in combination with modern immunotherapeutics.^[1]^ So far, only three platinum complexes have been clinically approved for worldwide use (cisplatin, carboplatin and oxaliplatin), while five more are applied in Asia exclusively.^[2]^ The mode of action involves hydrolysis of the leaving ligands inside the cell and subsequent binding to guanine and adenine of the DNA, thus inducing apoptosis.^[3]^ However, these processes can also occur in healthy tissues resulting in a variety of dose‐limiting side effects.^[4]^ One possibility to reduce this problem is the use of platinum(IV) complexes, as these oxidized counterparts are kinetically more stable, preventing many side reactions. Furthermore, the two introduced axial ligands can be utilized for targeting, attachment of bioactive ligands or the fine‐tuning of physico‐chemical properties.^[5]^

Different prerequisites exist for axial and equatorial ligands: While the axial ligands are often used to implement additional functionalities into the platinum(IV) complexes and should be stable against hydrolysis, the equatorial “leaving group” ligands should hydrolyze once the complex is reduced to platinum(II) inside the cancer cell. Nevertheless, some research groups synthesized and compared complexes with e.g. dichloroacetate,^[6]^ acetate,^[7]^ or ethacrynic acid,^[8]^ coordinated as axial ligands to platinum(IV) and as equatorial ligands to platinum(II), respectively. These data suggest that the equatorial plane can also be utilized for direct attachment of bioactive ligands.

**Scheme 1:**
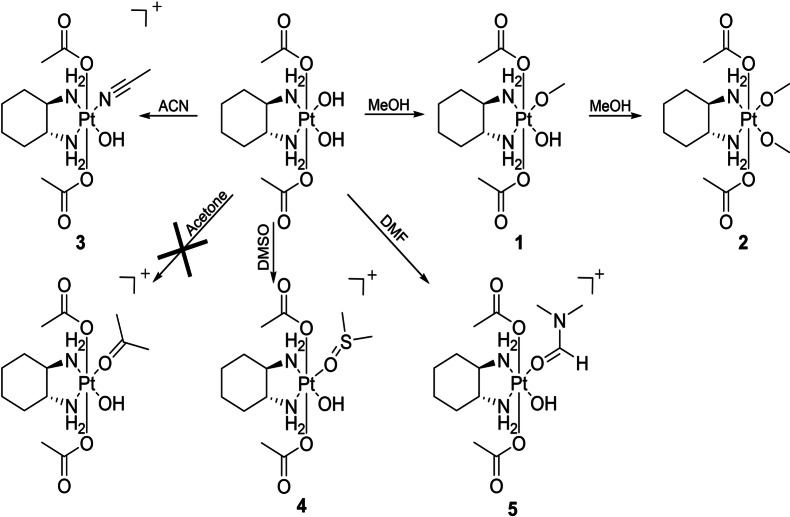
Overview of the reactions of [(DACH)Pt(OH)_2_(OAc)_2_] with different solvents.

Recently, we reported on the unexpected hydrolysis of the equatorial “leaving group” of certain platinum(IV) complexes under physiological conditions. We could show that oxali‐ and satraplatin(IV) complexes hydrolyze rapidly, while the respective carboplatin(IV) complex was completely stable and the cisplatin(IV) analogue was in between. This also enabled the synthesis of a novel oxaliplatin(IV) complex bearing two equatorial hydroxido ligands: [(DACH)Pt(OH_eq_)_2_(OAc_ax_)_2_] (DACH=(1*R*,2*R*)‐1,2‐diaminocyclohexane; Scheme 1).^[9]^ The question arose, if this complex could be used as synthetic precursor, enabling the direct and selective introduction of novel equatorial ligands into platinum(IV) complexes. In literature only very few publications with tetra‐hydroxidoplatinum(IV) complexes exist, where such substitutions have been reported.^[10]^ However, the presence of four hydroxido groups, two axial and two equatorial, does not allow for specific reactions in the equatorial plane, resulting in isomeric mixtures,^[10c]^ or the same ligand in all four positions.^[10b]^

Conventionally, the development of platinum(IV) complexes starts with the synthesis of the respective platinum(II) core, which is then oxidized e.g. with hydrogen peroxide to introduce two axial hydroxido groups, which can subsequently be functionalized to esters, carbonates or carbamates.^[11]^ In addition, few examples of axial halido ligand exchange reactions have been recently reported to introduce e.g. axial N‐heterocycles.^[12]^ Selective substitution of the equatorial “leaving group” in platinum(IV) complexes would be an elegant new synthetic pathway. Moreover, the oxidation step is not suitable for every ligand, as some of them might be prone to oxidation themselves. One example is biotin, for which the thioether can be oxidized into a sulfoxide or even a sulfone.^[13]^ Furthermore, such sulfur atoms also have a high affinity for direct coordination to the “soft” platinum(II) center; which is prevented when introducing the ligand in the “hard” platinum(IV) oxidation state. Biotin is an important cofactor in a variety of enzymes related to gluconeogenesis or fatty acid synthesis.^[14]^ It is taken up into the cell by the sodium‐dependent multivitamin transporter (SMVT), which is overexpressed in many types of cancer, and has therefore been suggested as a targeting moiety.^[15]^ Biotin was already investigated as an axial ligand for cisplatin(IV) complexes, showing promising results.^[16]^

Herein, we chose biotin for the establishment of the equatorial substitution reaction using the specific properties of [(DACH)Pt(OH_eq_)_2_(OAc_ax_)_2_], generating complexes that are not accessible by conventional synthetic routes. Subsequently, their hydrolytic and reductive behavior was compared with analogues bearing biotin in axial position. Furthermore, the biological activity against cancer cells was analyzed, indicating that equatorial substitution of platinum(IV) drugs results in reduced cytotoxicity. This renders such complexes ideal tools for prodrug concepts as shown by the synthesis of the first equatorial maleimide‐bearing platinum(IV) complexes (also not possible to be obtained by standard synthetic methods) for albumin‐mediated drug delivery.

## Results and Discussion

In the first step, we wanted to understand if the two equatorial hydroxido groups of [(DACH)Pt(OH_eq_)_2_(OAc_ax_)_2_] are suitable for substitution reactions and can be used for new synthetic strategies. Therefore, [(DACH)PtOx(OAc)_2_] (Ox=oxalate) was stirred in aqueous solution at pH 9 for 24 h leading to the release of the oxalate ligand and formation of [(DACH)Pt(OH_eq_)_2_(OAc_ax_)_2_].^[9]^ Subsequently, the isolated complex was incubated for 24 h at room temperature (RT) with different solvents (acetone, acetonitrile (ACN), dimethylformamide (DMF), dimethyl sulfoxide (DMSO), methanol (MeOH)) (Scheme 1). The reactions were monitored via high‐performance liquid chromatography coupled to a mass spectrometer (HPLC‐MS).

In MeOH, [(DACH)Pt(OH)_2_(OAc)_2_] formed the mono‐ and bis‐methanolato complexes **1** and **2**. This is in agreement with the X‐ray crystal structure of [(DACH)Pt(OH)_2_(OAc)_2_] from MeOH/diethyl ether, where also both hydroxido ligands were exchanged by MeOH.^[9]^ In ACN, exclusively the mono‐ACN complex **3** was formed. In contrast, acetone did not coordinate to platinum, instead again the mono‐ACN complex **3** could be observed due to the ACN in the HPLC solvent (also direct injection of the acetone solution into the MS did not show coordination to platinum). In case of DMSO and DMF, also the respective mono‐solvent complexes **4** and **5** were formed. Comparing all tested solvents, MeOH coordinated most readily and was the only solvent resulting in the formation of a bis‐species.

The stability of the formed complexes in aqueous solution at physiological pH was investigated after incubation of 100 μM [(DACH)Pt(OH)_2_(OAc)_2_] in the different solvents for 24 h and drying of the samples under reduced pressure. The remaining solids were taken up in phosphate buffer at pH 7.4 and tested for their stability against hydrolysis with HPLC‐MS. The mono‐ACN and ‐DMF complexes **3** and **5** hydrolyzed quickly, again forming [(DACH)Pt(OH)_2_(OAc)_2_] after 1 h. The MeOH complexes **1** and **2** showed slower hydrolysis of their methoxido ligands (Figure S2), with a rate comparable to the oxalate release from [(DACH)PtOx(OAc)_2_].^[9]^ The sample dissolved in DMSO turned black over the course of 24 h. As a result, it could not be tested for hydrolytic stability. However, further examination of the HPLC‐MS runs revealed the formation of the tetra‐acetato complex, which could also be isolated via preparative HPLC (data not shown). This suggests that axial acetate ligands, which are released during decomposition/reduction, can coordinate to still intact molecules of [(DACH)Pt(OH)_2_(OAc)_2_] (or displace a DMSO ligand). Consequently, already quite low levels of carboxylic acids (e.g. acetate) seem to be sufficient to replace hydroxido ligand(s) in [(DACH)Pt(OH)_2_(OAc)_2_]. Therefore, we investigated the reaction of [(DACH)Pt(OH)_2_(OAc)_2_] dissolved in acetone (where the complex is stable) and 2–250 eq. of acetic acid for 24 h at RT. Even with only 10 eq. full conversion to the tetra‐acetato complex in an analytical scale was observed. Therefore, we scaled up and optimized this reaction (Table 1).

**Table 1:** Overview on the reaction conditions and yields of [(DACH)Pt(OH)_2_(OAc)_2_] and different ligands. 10 eq. of ligand were added in all cases.

Ligand	Temp	Solvent	[(DACH)Pt(OH)_2_(OAc)_2_] [mmol/l]	Yield^[b]^
Acetic acid	RT^[a]^	Acetone	7.2	40 % Mono+ 20 % Bis
Acetic acid	RT	Water	7.2	no reaction
Acetic acid	RT	DMF	7.2	40 % Mono+ 20 % Bis
Sodium oxalate	RT	DMF	7.2	10 % Mono+ 20 % Bis
Oxalic acid	RT	DMF	7.2	10 % Mono+ 30 % Bis
Biotin	RT	DMF	7.2	30 % Mono+ 0 % Bis
Biotin	RT	DMF	27	20 % Mono+ 20 % Bis
Biotin	50 °C	DMF	27	30 % Mono+ 60 % Bis

[a] RT=room temperature; [b] yield determined via HPLC‐MS after 24 h reaction time.

All reactions were carried out with 5 mg of [(DACH)Pt(OH)_2_(OAc)_2_], 10 eq. of the respective ligand and a reaction time of 24 h. First, the reaction with acetic acid in different solvents was tested: in water the reaction did not take place at all, which is also in agreement with the high stability of [(DACH)Pt(OH)_2_(OAc)_2_] in medium and serum.^[9]^ In contrast, in both acetone and DMF approx. 60 % of the di‐hydroxido complex reacted to the mono‐ or bis‐acetato species. Hence, further experiments were conducted in DMF, being the more versatile solvent. Of note, using [(DACH)PtCl_2_(OAc)_2_] as the precursor under the same conditions did not result in any conversion. Next, it was investigated if [(DACH)Pt(OH)_2_(OAc)_2_] could be transformed back to its original oxaliplatin core. Addition of both sodium oxalate and oxalic acid resulted in the desired complex (20 % and 30 %, respectively). This comparison furthermore revealed that acids might be preferred in this type of reaction. Finally, the biologically active targeting ligand biotin was used. While under the “standard” conditions only the mono‐complex was formed, the bis‐complex could be obtained in good yields (60 %) by 4‐fold higher concentrated solution and 50 °C reaction temperature. Under these new conditions the mono‐ and bis‐complexes formed even with only one (10 % mono; 5 % bis) or two eq. of biotin (5 % mono; 15 % bis). Of note, when using 2.5 eq. biotin and 2.5 eq. 2‐(1H‐benzotriazole‐1‐yl)‐1,1,3,3‐tetramethylaminium tetrafluoroborate (TBTU) as coupling agent exclusively the bis‐complex was formed in 30 % yield. Consequently, when only limited amounts of the carboxylate ligand are available, this could be an interesting alternative strategy.

As proof of principle, we investigated if equatorial ligand exchange is also possible for a cisplatin core, which is generally much more resistant against equatorial hydrolysis.^[9]^ Therefore, we further increased the pH value and temperature, and incubated [(NH_3_)_2_PtCl_2_(OAc)_2_] in aqueous phosphate buffered solution at pH 10 at 50 °C, resulting in a mixture of 80 % [(NH_3_)_2_Pt(OH_eq_)_2_(OAc_ax_)_2_] and 20 % [(NH_3_)_2_PtOH_eq_Cl_eq_(OAc_ax_)_2_] after 24 h. The obtained (OH_eq_)_2_ species was then incubated with 10 eq. biotin at 50 °C for 24 h, resulting in exclusive formation of the biotin/OH complex (even after 48 h reaction time). Notably, when incubating the equatorially mixed OH/Cl species [(NH_3_)_2_PtOH_eq_Cl_eq_(OAc_ax_)_2_], the OH was exchanged at the same rate as for the (OH_eq_)_2_ compound, resulting in the biotin/Cl complex. Consequently, also cisplatin(IV) species can be used for equatorial ligand substitution reactions.

**Scheme 2:**
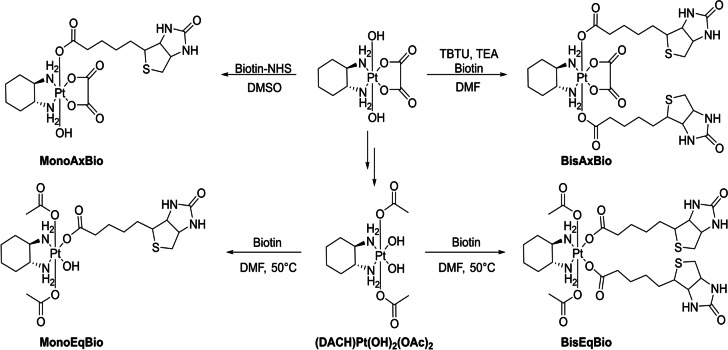
Structures of synthesized biotin‐platinum(IV) complexes.

However, our focus was to compare the properties of equatorially substituted complexes with the respective axial counterparts. Therefore, the following oxaliplatin(IV) panel was synthesized (Scheme 2): the equatorial mono‐biotin (**MonoEqBio**) and bis‐biotin complex (**BisEqBio**), and as axial references the oxaliplatin(IV) complexes with one (**MonoAxBio**) or two biotin ligands (**BisAxBio**). For purification of **MonoEqBio** on the preparative HPLC, pure solvents had to be used, as the usually added 0.1 % formic acid or trifluoroacetic acid (TFA) could replace the OH at the platinum core. The purity of all complexes was confirmed via NMR, MS and elemental analysis. Efforts to synthesize the respective biotin‐platinum(II) reference complexes were not successful. Most probably, the soft platinum(II) readily coordinates the thioether of biotin resulting in various different species.

**Figure 1:**
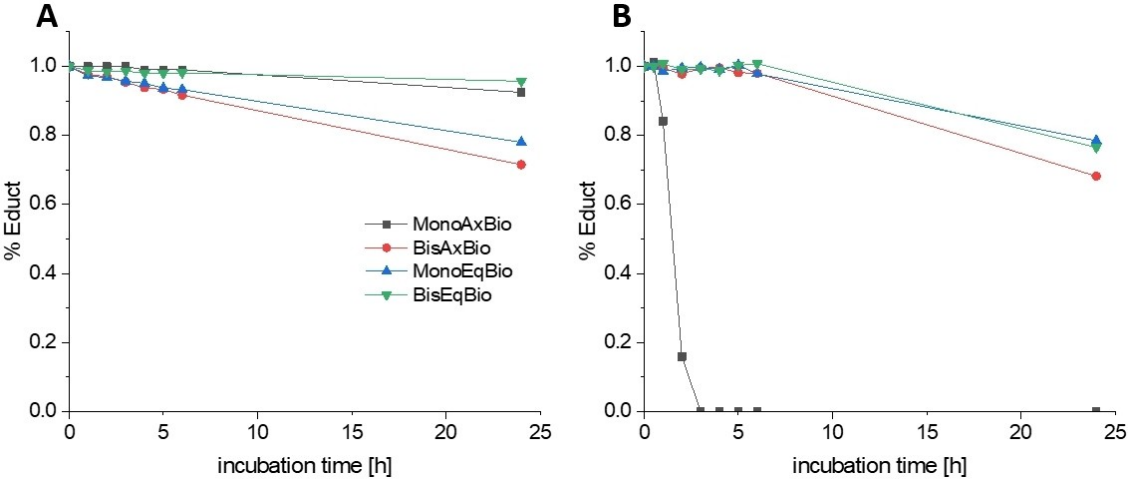
A) Equatorial hydrolysis of the investigated platinum complexes at 37 °C (1 mM complex in 100 mM phosphate buffer pH 7.4). B) Reduction kinetics of the investigated platinum complexes at 20 °C (1 mM complex with 10 eq. of ascorbic acid in 150 mM phosphate buffer pH 7.4).

In order to compare the properties of the different oxaliplatin(IV)‐like biotin complexes (Scheme 2), first the hydrolytic stability of the equatorial ligands was evaluated. The complexes (1 mM) were incubated in 100 mM phosphate buffer (pH 7.4) at 37 °C (Figure 1A). While **MonoAxBio** was quite stable against equatorial oxalate hydrolysis (8 % mono‐OH species after 24 h), **BisAxBio** showed substantial parts of hydrolyzed species after 24 h of incubation (24 % mono‐hydroxido; 4 % di‐hydroxido). The high stability of the **MonoAxBio** derivative compared to the **BisAxBio** complex could be confirmed by the reference complexes [(DACH)PtOxOH_ax_OAc_ax_] and [(DACH)PtOx(OAc)_2_], showing 2 % and ≈50 % equatorial hydrolysis at the platinum(IV) center after 24 h, respectively (Figure S3). This trend was reversed for the complexes with equatorially bound biotin: **BisEqBio** showed only minimal hydrolysis (4 % after 24 h), while 22 % of **MonoEqBio** were hydrolyzed to the di‐hydroxido species after 24 h, which also allowed the detection of released biotin (Figure S4). This data shows that regarding equatorial hydrolysis of platinum(IV) complexes the two directly adjacent carboxylic acid groups in the oxalate ligand of **BisAxBio** have a distinctly lower coordination strength, when compared to the two monodentate carboxylate ligands in **BisEqBio**. In line, the carboplatin analogue [(DACH)Pt(CBDCA)(OAc)_2_] (CBDCA=1,1‐cyclobutanedicarboxylic acid) with one carbon atom between the bidentate carboxylates was very stable (2 % hydrolysis after 24 h; data not shown) indicating that **BisEqBio** behaves more like a carboplatin than an oxaliplatin derivative.

The reduction kinetics of the complexes in the presence of 10 eq. ascorbic acid at 20 °C (Figure 1B) revealed that **MonoAxBio** was completely reduced after 3 h, in accordance to literature on other platinum(IV) complexes with one axial OH group.^[17]^ The other three complexes were much more stable, with **MonoEqBio** and **BisEqBio** at ≈80 % intact after 24 h and **BisAxBio** at ≈70 % (again, for the axial biotin complexes **MonoAxBio** and **BisAxBio** the release of biotin and oxaliplatin could be confirmed; Figure S5). Interestingly, the free OH moiety in equatorial position (**MonoEqBio**) did not accelerate the reduction, as was observed for the axial analogue **MonoAxBio**.

**Figure 2:**
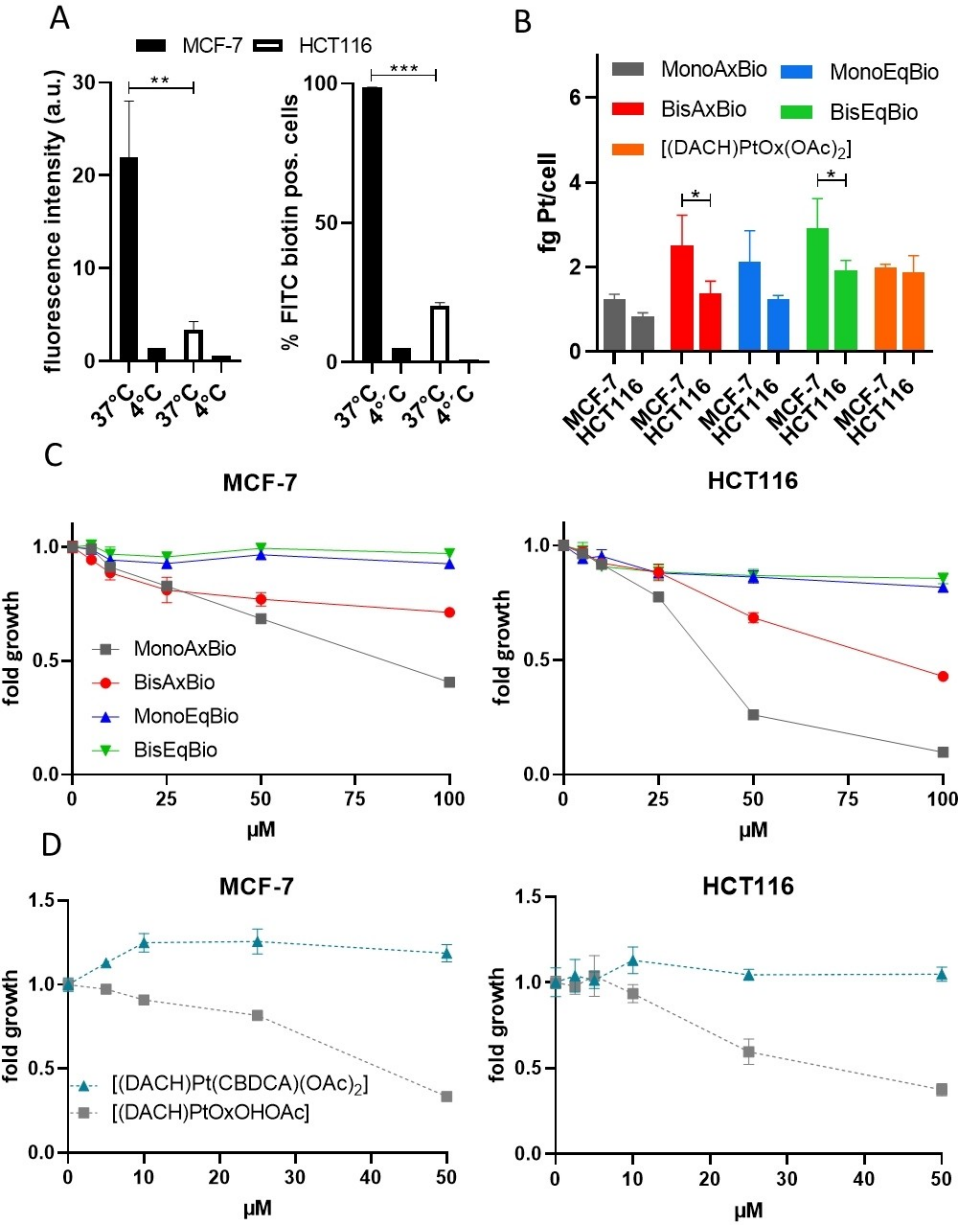
Characterization of the cellular biotin and drug uptake and anticancer activity of the platinum(IV) complexes in MCF‐7 (SMVT high) and HCT116 cells (SMVT low). A) Cells were incubated with FITC‐labeled biotin (20 μM) at 37 °C or 4 °C for 5 h. Mean FITC fluorescence intensity (left) FITC‐positive cells (right) were determined by flow cytometry (ex/em: 488/530 nm, fluorescence intensity was normalized to auto fluorescence control). B) Cellular uptake of indicated drugs (10 μM) was determined by ICP‐MS after 3 h incubation. Values in (A) and (B) are presented as means ± SD of three independent experiments. Statistical significance was tested by one‐way ANOVA and Dunnett's multiple comparison test (*p<0.05, **p<0.01 and ***p<0.001). (C) Anticancer activity of biotin‐containing platinum compounds and platinum reference compounds after 72 h was determined via MTT‐based assays. In case of (C) and (D), values refer to means±SD of one representative experiment performed in triplicates.

To test the impact of the equatorial/axial ligand coordination on the cellular drug uptake, two cancer cell models with different SMVT expression, indicative for their biotin uptake ability, were employed (human colorectal cancer HCT116 and human breast cancer MCF‐7 cells).^[18]^ The superior and active biotin uptake of MCF‐7 cells (compared to HCT116 cells) was first confirmed by flow cytometry after incubation with FITC‐labeled biotin (Figure 2A). Subsequently, inductively coupled plasma mass spectrometry (ICP‐MS) was used to detect the intracellular platinum levels after treatment with the new biotin‐targeted platinum(IV) derivatives. [(DACH)PtOx(OAc)_2_] was used as a reference. As shown in Figure 2B, especially in case of the two bis‐biotin drugs, the SMVT‐high MCF‐7 cells had significantly higher platinum uptake than SMVT‐low HCT116 cells. A similar pattern was observed for the mono‐biotin drugs (although it did not reach statistical significance). In contrast, the drug uptake of [(DACH)PtOx(OAc)_2_] was not influenced by the SMVT expression levels.

To test the anticancer activity of the complexes, we first compared the impact of free oxaliplatin and [(DACH)PtOx(OAc)_2_] in MCF‐7 and HCT116 cells after 72 h via MTT‐based assays (Figure S6). Noteworthy, oxaliplatin was distinctly more active against HCT116 (IC_50_ of 1.88 μM) than MCF‐7 cells (IC_50_>10 μM). This is in good agreement with oxaliplatin being the standard therapy against advanced colorectal cancer.^[19]^ Also in case of [(DACH)PtOx(OAc)_2_], the anticancer activity was more pronounced against HCT116 than MCF‐7 cells (16.8 μM vs 34.4 μM, respectively), but in general distinctly less active due to the prodrug nature of the platinum(IV) complex.^[20]^ The four biotin‐conjugated platinum(IV) compounds (Scheme 2) revealed strong differences under the same conditions (Figure 2C). The two equatorially biotin‐conjugated substances showed no activity up to the highest concentration of 100 μM, **BisAxBio** was slightly cytotoxic and **MonoAxBio** was the most active derivative. Overall, the general pattern and activity of the tested drugs was similar in both cell models (especially interesting considering the reduced sensitivity of MCF‐7 to oxaliplatin) indicating that the differences in anticancer activity cannot originate from the different biotin uptake efficiency. The higher activity of **MonoAxBio** can be explained by the very fast reduction compared to the other derivatives (Figure 1B). Accordingly, viability experiments with the reference complex [(DACH)PtOxOHOAc] revealed similar anticancer activity (Figure 2D). However, that **MonoEqBio** and **BisEqBio**, bearing monodentate biotin ligand(s), were even less active than **BisAxBio** with a bidentate oxalate ligand was unexpected. As the reduction properties of these three complexes are quite similar (Figure 1B), we hypothesized that the underlying reason has to be the hydrolysis rate of the formed platinum(II) complexes. Indeed, subsequently performed viability experiments with the carboplatin reference [(DACH)Pt(CBDCA)(OAc)_2_] showed activity comparable to the equatorial‐biotin complexes (Figure 2D). This suggests, that the specific reduction and hydrolysis characteristics of the individual derivative is in fact crucial for their anticancer activities.

Noteworthy, prolonged drug incubation (10 days clonogenicity tests) not only enhanced the general activities of the drugs (due to longer time for prodrug and platinum(II) activation), but also inverted the response pattern of the equatorial drugs (Figure S7). Thus, the drugs with equatorial biotin were now more active against the oxaliplatin‐resistant SMVT‐high MCF‐7 than HCT116 cells. In contrast, the axial‐conjugated biotin drugs showed an activity pattern comparable to the 72 h experiments. This indicates that the exact position of the ligands can distinctly influence the activity as well as the (platinum) resistance pattern of the complexes.

Taken together the data clearly show that **MonoEqBio** and **BisEqBio** are highly stable platinum(IV) complexes, which after reduction generate platinum(II) derivatives with slow ligand release more comparable to carboplatin than oxaliplatin. Consequently, we hypothesized that these properties of equatorial monodentate carboxylate ligands are ideal for the use of long‐circulating drug delivery systems, where slow release/activation is desired. Miriplatin emulsions^[21]^ (approved in Japan against hepatocellular carcinoma) or liposomal aroplatin^[22]^ (studied up to clinical phase II) are representatives of such type of platinum(II) complexes with two monodentate carboxylate ligands. One elegant drug delivery strategy is the use of albumin as a drug carrier via maleimide chemistry. We recently developed platinum(IV) complexes with axial maleimide ligand(s).[^20b^, ^23^] However, introduction of the albumin‐targeting maleimide at the equatorial position(s) would allow the attachment of two (different) synergistic drugs at the free axial positions, generating targeted triple‐action prodrugs within one platinum complex.

**Figure 3:**
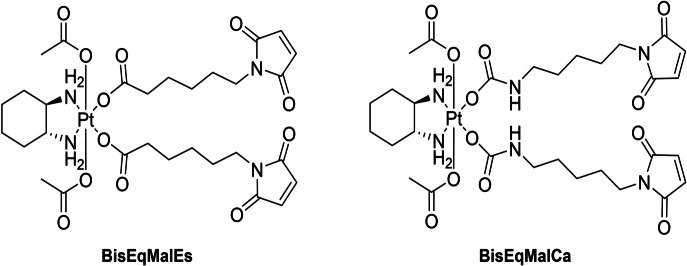
Structures of synthesized maleimide‐bearing platinum(IV) complexes.

We therefore synthesized two different equatorial maleimide‐platinum(IV) complexes (Figure 3) following the procedure described above. **BisEqMalEs** was synthesized by incubation of 6‐maleimidehexanoic acid and [(DACH)Pt(OH)_2_(OAc)_2_], whereas for the carbamate analogue (**BisEqMalCa**) the maleimide isocyanate was used. Of note, such maleimide‐bearing complexes could not be synthesized from the respective platinum(II) precursors by conventional synthetic methods, as already the oxidation with H_2_O_2_ leads to maleimide hydrolysis. To the best of our knowledge **BisEqMalCa** is also the first platinum(IV) complex with equatorial carbamate leaving groups. Incubation of the complexes at pH 7.4 revealed high stability (Figure S8), except for the well‐known maleimide hydrolysis at physiological pH (Figure S9).^[24]^ This phenomenon also hampered a quantitative evaluation of the reduction rate as both processes are overlaying; nevertheless similar reduction rates could be observed for **BisEqMalEs** and **BisEqMalCa** (Figure S10). In addition, albumin‐binding studies via size exclusion chromatography (SEC) coupled to ICP‐MS, showed fast binding rates for both complexes in fetal calf serum (FCS, buffered with 150 mM phosphate buffer to ensure a stable pH) at 37 °C (Figure S11).

**Figure 4:**
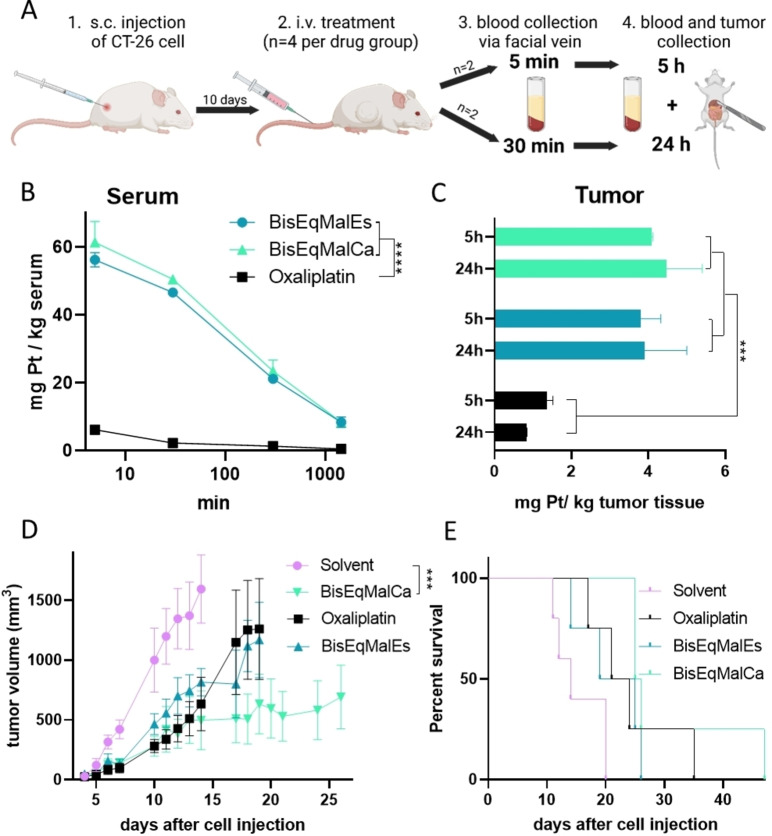
Pharmacological evaluation and anticancer activity of the platinum complexes in CT‐26‐bearing Balb/c mice. For the pharmacological studies, animals were treated once i. v. with concentrations equimolar to 9 mg/kg oxaliplatin. A) treatment and sample collection scheme for pharmacological studies. B) serum samples were collected after 5 min, 30 min, 300 min and 1440 min; C) tumor samples were collected after 5 h and 24 h. Platinum levels of all samples were measured via ICP‐MS. In case of the evaluation for the anticancer activity, CT‐26‐bearing Balb/c mice were treated twice a week for two weeks i. v. with concentrations equimolar to 9 mg/kg oxaliplatin. (D) Impact on tumor growth; data are presented as means±SEM. (E) The overall survival is depicted via a Kaplan–Meier curve. Statistical significance for (B) and (D) was tested by two‐way ANOVA (*** p<0.001). The oxaliplatin data for the pharmacological analysis were used from Schueffl et al.^[20b]^

Evaluation of maleimide complexes in cell culture is usually difficult, due to the reaction with the artificially high content of amino acids in the culture medium. Consequently, the two new albumin‐binding compounds were directly tested for their pharmacokinetic properties in vivo using CT‐26 colon cancer‐bearing Balb/c mice (Figure 4A). Indeed, the maleimide‐functionalization not only resulted in a distinctly prolonged plasma half‐life time, but also in enhanced drug accumulation in the tumor tissue compared to free oxaliplatin (Figure 4B&C), in good agreement with axial maleimide‐containing platinum(IV) derivatives.[^20b^, ^25^] This also resulted in superior anticancer activity of **BisEqMalCa** compared to oxaliplatin (Figure 4D). Of note, this was not the case for **BisEqMalEs**. Interestingly, a distinctly higher activity of the “carbamate‐linked” maleimide compared to the “ester‐linked” analog was recently also observed for axial albumin‐targeting platinum(IV) drugs,^[25]^ although the underlying reasons are so far unknown.

## Conclusion

Taken together, this work presents a new strategy for the synthesis of platinum(IV) complexes, with introduction of the equatorial ligands as the final step. This especially enables the insertion of sensitive ligands, which do not survive the platinum(II) oxidation process using H_2_O_2_. The equatorial biotin ligands of the newly synthesized complexes **MonoEqBio** and **BisEqBio** showed very slow release kinetics (comparable to carboplatin), ideal for long‐circulating drug delivery strategies. Indeed, two complexes with equatorially inserted maleimide ligands (**BisEqMalEs** and **BisEqMalCa**) for endogenous albumin‐binding revealed greatly improved pharmacokinetic properties and tumor accumulation in mice. **BisEqMalCa** also showed distinctly higher anticancer activity compared to the approved reference oxaliplatin. Shifting the drug‐targeting moiety from the typical axial position into the equatorial plane generates two free axial positions for attachment of (different) synergistic drugs, which will enable the development of new types of triple‐action platinum(IV) prodrugs.

## Conflict of interest

The authors declare no conflict of interest.

1

## Supporting information

As a service to our authors and readers, this journal provides supporting information supplied by the authors. Such materials are peer reviewed and may be re‐organized for online delivery, but are not copy‐edited or typeset. Technical support issues arising from supporting information (other than missing files) should be addressed to the authors.

Supporting Information

## Data Availability

The data that support the findings of this study are available from the corresponding author upon reasonable request.
